# Constraints on Non-Flat Starobinsky *f*(*R*) Dark Energy Model

**DOI:** 10.3390/e23101320

**Published:** 2021-10-10

**Authors:** Chao-Qiang Geng, Yan-Ting Hsu, Jhih-Rong Lu

**Affiliations:** 1 School of Science, Chongqing University of Posts & Telecommunications, Chongqing 400065, China; 2 Department of Physics, National Tsing Hua University, Hsinchu 300, Taiwan; ythsu@gapp.nthu.edu.tw (Y.-T.H.); jhih-ronglu@gapp.nthu.edu.tw (J.-R.L.); 3 Synergetic Innovation Center for Quantum Effects and Applications (SICQEA), Hunan Normal University, Changsha 410081, China

**Keywords:** modified gravitational theory, non-flat spacetime

## Abstract

We study the viable Starobinsky f(R) dark energy model in spatially non-flat FLRW backgrounds, where $f\left(R\right)=R-\lambda R_{ch}\left[1-{\left(1+R^{2}/R_{ch}^{2}\right)}^{-1}\right]$ with $R_{ch}$ and λ representing the characteristic curvature scale and model parameter, respectively. We modify CAMB and CosmoMC packages with the recent observational data to constrain Starobinsky f(R) gravity and the density parameter of curvature $\Omega_{K}$. In particular, we find the model and density parameters to be $\lambda^{-1}<0.283$ at 68% C.L. and $\Omega_{K}=-0.00099_{-0.0042}^{+0.0044}$ at 95% C.L., respectively. The best $\chi^{2}$ fitting result shows that $\chi_{f(R)}^{2}≲\chi_{\Lambda CDM}^{2}$, indicating that the viable f(R) gravity model is consistent with ΛCDM when $\Omega_{K}$ is set as a free parameter. We also evaluate the values of AIC, BIC and DIC for the best fitting results of f(R) and ΛCDM models in the non-flat universe.

## 1. Introduction

It is known that cosmological observations, such as Supernova type Ia [1,2], Planck [3,4,5,6], and BAO [7], have provided strong evidence that our current universe is accelerating. Among the numerous attempts to describe this late time accelerating epoch of the universe, ΛCDM is the most successful and simplest one. However, it still confronts some unsolved issues, such as the cosmological constant (CC) problem [8]. This CC problem has motivated people to search for various new theories beyond ΛCDM, such as f(G) [9,10,11], scale dependence cosmology [12,13,14], and scalar tensor [15,16] theories. A typical model of such theories is the f(R) gravity theory, in which the Ricci scalar of *R* in the Einstein–Hilbert action of the standard general relativity (GR) is modified to an arbitrary function of f(R) [17,18,19,20,21,22,23].

Among f(R) gravity theories, the Starobinsky f(R) dark energy model [24] is one of the models that satisfy all the viable conditions, which include (1) positivity of effective gravitational coupling constant, resulting in $f_{R}>0$; (2) stability of cosmological perturbations, leading to $f_{RR}>0$; (3) an asymptotic behavior to the ΛCDM model in the large curvature region; (4) a late-time stable de-Sitter solution; and (5) solar system constraints. The Starobinsky f(R) model takes the form [24]:

(1) $$\begin{array}{c} f\left(R\right)=R-\lambda R_{ch}\left[1-{\left(1+\frac{R^{2}}{R_{ch}^{2}}\right)}^{-n}\right]\;, \end{array}$$

where λ and *n* are the dimensionless model parameters, and $R_{ch}$ is the characteristic curvature. The model has a feature that it contains a “disappearing" cosmological constant when curvature is negligible, i.e., R→0. That is, in such a model, the effects of dark energy could be understood as a pure geometrical effect and has little to do with the quantum vacuum energy [24]. It has been shown that this is a curvature singularity problem in viable f(R) gravity [25,26], and it has been proposed that if an additional $R^{n}$ term with 1<n≤2 is introduced [27,28,29,30], the singularity can be avoided.

In addition, there is evidence from the Planck2018 CMB data along with ΛCDM that our universe is closed in 99% C.L. [31]. This motivates us to investigate whether the universe is also a spatially curved one if we assume a model from modified gravity rather than ΛCDM. In this study, we will focus on the viable Straobinsky f(R) model and modify the CAMB and CosmoMC packages at the background level.

The paper is organized as follows. In Section 2, we review the Friedmann equations in f(R) gravity in the non-flat backgrounds. In Section 3, we present the evolutions of $\rho_{DE}/\rho_{DE}^{0}$ and $w_{DE}$ for the Starobinsky f(R) model in a flat and non-flat universe, respectively. We also constrain the model parameters by using the Markov Chain Monte Carlo (MCMC) method. We summarize our results in Section 4.

## 2. Starobinsky *f*(*R*) Gravity in the Non-Flat Universe

The action of f(R) gravity is given by

(2) $$\begin{array}{c} S=\int d^{4}x\frac{\sqrt{-g}}{2\kappa^{2}}f\left(R\right)+S_{M}, \end{array}$$

where $\kappa^{2}=8\pi G$ with *G* as the Newton’s constant, $S_{M}$ is the action for both relativistic and non-relativistic matter. The field equations can be obtained by varying the action (2), given by

(3) $$\begin{array}{c} G_{\mu \nu }=\kappa^{2}\left(T_{\mu \nu }^{\left(M\right)}+T_{\mu \nu }^{\left(de\right)}\right)\;, \end{array}$$

where $G_{\mu \nu }=R_{\mu \nu }-\left(1/2\right)g_{\mu \nu }R$ is the Einstein tensor, $T_{\mu \nu }^{\left(M\right)}$ represents the energy-momentum tensor for relativistic and non-relativistic matter, and

(4) $$\begin{array}{c} T_{\mu \nu }^{\left(de\right)}=\frac{1}{\kappa^{2}}\left(G_{\mu \nu }-FR_{\mu \nu }+\frac{1}{2}g_{\mu \nu }f+\nabla_{\mu }\nabla_{\nu }F-g_{\mu \nu }□F\right) \end{array}$$

with F≡df(R)/dR and $□\equiv g^{\mu \nu }\nabla_{\mu }\nabla_{\nu }$ the d’Alembert operator.

To describe our universe, we consider homogenous and isotropic Friedmann–Lemaitre–Robertson–Walker (FLRW) spacetime, given by

(5) $$ds^{2}=-dt^{2}+a^{2}\left(t\right)\left(\frac{dr^{2}}{1-Kr^{2}}+r^{2}d\theta^{2}+r^{2}sin^{2}\theta d\phi^{2}\right),$$

where a(t) is the scale factor, and K=+1,0,−1 correspond to the spatially closed, flat and open universe, respectively. With Equations (3) and (5), one is able to obtain the modified Friedmann equations as:

(6) $$\begin{array}{cc} H^{2} & =\frac{\kappa^{2}}{3}\left(\rho_{M}+\rho_{DE}+\rho_{K}\right), \end{array}$$

(7) $$\begin{array}{cc} \dot{H} & =-\frac{\kappa^{2}}{2}\left(\rho_{M}+\rho_{DE}+\rho_{K}+P_{M}+P_{DE}+P_{K}\right), \end{array}$$

where $\rho_{M}=\rho_{m}+\rho_{r}$ is the density of non-relativistic matter and radiation, while the dark energy density and pressure are given by

(8) $$\begin{array}{cc} \rho_{DE} & =\frac{3}{\kappa^{2}}\left(H^{2}\left(1-F\right)-\frac{1}{6}\left(f-FR\right)-H\dot{F}+\frac{K}{a^{2}}\left(1-F\right)\right), \end{array}$$

(9) $$\begin{array}{cc} P_{DE} & =\frac{1}{\kappa^{2}}\left(\ddot{F}+2H\dot{F}+\frac{1}{2}\left(f-FR\right)-\left(1-F\right)(3H^{2}+2\dot{H}+\frac{K}{a^{2}})\right), \end{array}$$

respectively. Note that the effects of spatial curvature in the modified Friedmann equations can be described by the effective curvature energy density and pressure, written as

(10) $$\begin{array}{cc} \rho_{K} & =-\frac{3K}{\kappa^{2}a^{2}}, \end{array}$$

(11) $$\begin{array}{cc} P_{K} & =\frac{K}{\kappa^{2}a^{2}}, \end{array}$$

respectively. Furthermore, to solve the modified Friedmann equations numerically, we define the dimensionless parameter $y_{H}$ to be

(12) $$\begin{array}{cc} y_{H} & \equiv \frac{\rho_{DE}}{\rho_{m}^{\left(0\right)}}=\frac{H^{2}}{m^{2}}-a^{-3}-\chi a^{-4}-\beta a^{-2}, \end{array}$$

where $m^{2}=\kappa^{2}\rho_{m}^{\left(0\right)}/3$, $\chi =\rho_{r}^{\left(0\right)}/\rho_{m}^{\left(0\right)}$, and $\beta =\rho_{K}^{\left(0\right)}/\rho_{m}^{\left(0\right)}$ with $\rho_{i}^{\left(0\right)}\equiv \rho_{i}\left(z=0\right)$. Consequently, one is able to rewrite Equation (6) in the following form, [28,32,33,34]

(13) $$\begin{array}{c} y_{H}^{\prime \prime }+J_{1}y_{H}^{\prime }+J_{2}y_{H}+J_{3}=0, \end{array}$$

where the prime “′” denotes the derivative w.r.t to lna, and

(14) $$\begin{array}{cc} J_{1} & =4+\frac{1}{y_{H}+a^{-3}+\chi a^{-4}+\beta a^{-2}}\frac{1-F}{6m^{2}F{,}_{R}}, \end{array}$$

(15) $$\begin{array}{cc} J_{2} & =\frac{1}{y_{H}+a^{-3}+\chi a^{-4}+\beta a^{-2}}\frac{2-F}{3m^{2}F{,}_{R}}, \end{array}$$

(16) $$\begin{array}{cc} J_{3} & =-3a^{-3}-\frac{\left(1-F\right)\left(a^{-3}+2\chi a^{-4}\right)+\left(R-f\right)/3m^{2}}{y_{H}+a^{-3}+\chi a^{-4}+\beta a^{-2}}\frac{1}{6m^{2}F{,}_{R}}. \end{array}$$

## 3. Numerical Results

We modified the CAMB [35] and CosmoMC [36,37] packages to study the cosmological evolutions and constraints of parameters for the Starobinsky f(R) model in the non-flat universe. We note that throughout this paper, we take the model parameter *n* in Equation (1) to be 1 in comparison with the previous study [38].

### 3.1. Cosmological Evolutions

We examine the evolutions of the normalized effective dark energy density $\rho_{DE}/\rho_{DE}^{0}$ and equation of state $w_{DE}$ for the Starobinsky f(R) model. In the previous study of the Starobinsky f(R) model with the flatness assumption [38], the model parameter is constrained to be $0.066<\lambda^{-1}<0.381$. In Reference [31], the spatial curvature density parameter is fitted to be $\Omega_{K}^{0}=0.00\pm 0.01$ at 68% C.L. In this work, we choose $\lambda^{-1}=0.4$ and $\Omega_{K}=\left(0.01,0,-0.01\right)$ for (open, flat, closed) universe to see the cosmological evolutions of Starobinsky f(R) gravity. To solve Equation (13) numerically, we integrate from the past ($z=z_{f}≃8.58,8.68,8.78$ for open, flat, and closed universe, respectively) to the present (z=0) and choose our initial condition at $z=z_{f}$ as [28]

(17) $$\begin{array}{cc} y_{H}\left(z_{f}\right) & =\frac{\Omega_{\Lambda }^{\left(0\right)}}{\Omega_{m}^{\left(0\right)}}, \end{array}$$

(18) $$\begin{array}{cc} \frac{dy_{H}\left(z\right)}{dz}{|}_{z=z_{f}} & =0, \end{array}$$

where $\Omega_{\Lambda }^{\left(0\right)}$ is the dark energy density parameter in ΛCDM and $m^{2}=\kappa^{2}\rho_{m}^{\left(0\right)}/3$. We have set $\Omega_{m}^{\left(0\right)}≃0.3144,\Omega_{\Lambda }^{\left(0\right)}≃\left(0.6742,0.6842,0.6942\right)$ for $\Omega_{K}^{\left(0\right)}=\left(0.01,0,-0.01\right)$. That is, we make the values of $y_{H}\left(z=z_{f}\right)$ in the Starobinsky f(R) model behave like $y_{H}\left(z=0\right)$ in ΛCDM. Note that the values of $\Omega_{m}^{\left(0\right)},\Omega_{\Lambda }^{\left(0\right)}≃0.6842$, and $\Omega_{K}^{\left(0\right)}=0$ are chosen according to the Planck 2018 Collaboration [4]. Here, we have used the fact that at a high redshift the universe should be very close to the ΛCDM model. Furthermore, as we want to examine the behavior of the Starobinsky f(R) model in the non-flat universe, we manually create two sets of initial conditions, $\Omega_{K}^{\left(0\right)}=\left(0.01,-0.01\right)$ and $\Omega_{DE}^{\left(0\right)}≃\left(0.6742,0.6942\right)$ for open and close universe, respectively.

**Figure 1:**
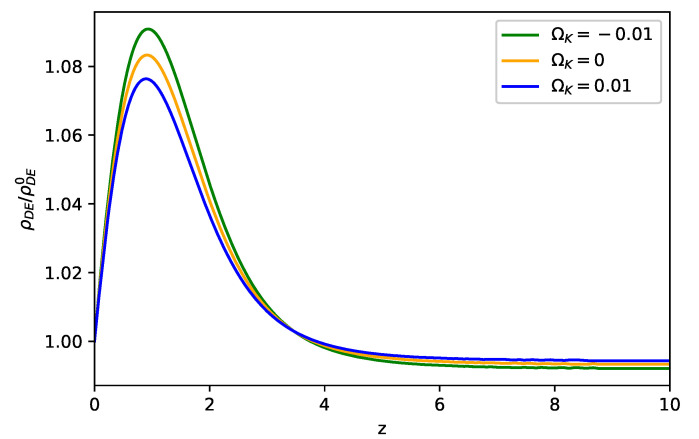
Evolutions of $\rho_{DE}/\rho_{DE}^{0}$ for the Starobinsky f(R) model with $\lambda^{-1}=0.4$ in the flat and non-flat universe, where $\rho_{DE}^{0}$ represents the energy density of dark energy at the present time, while the initial values are given by $\Omega_{m}^{\left(0\right)}≃0.3144$, and $\Omega_{\Lambda }^{\left(0\right)}≃\left(0.6742,0.6842,0.6942\right)$ for $\Omega_{K}^{0}=\left(0.01,0,-0.01\right)$.

**Figure 2:**
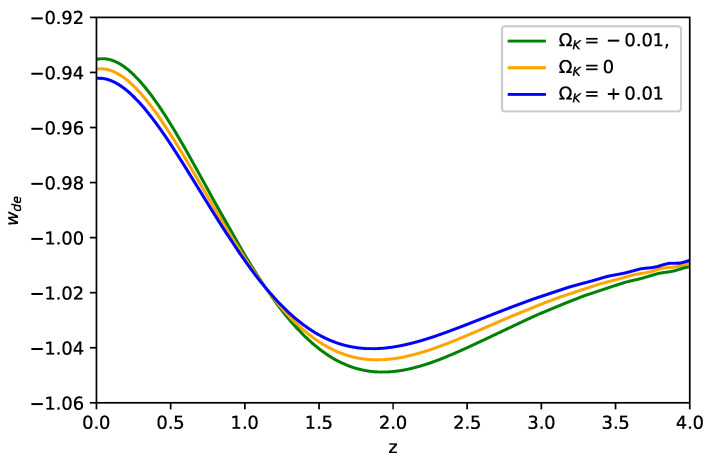
Evolutions of $w_{DE}$ for the Starobinsky f(R) model with $\lambda^{-1}=0.4$ in the flat and non-flat universe, where the initial values are the same as Figure 1.

As one of the features in the viable f(R) models, dark energy approaches the CC in the high redshift region, which can be seen in Figure 1. As shown in the figure, $\rho_{DE}/\rho_{DE}^{0}$ starts to evolve as z≲4 and approaches the maximum around z=1, where $\rho_{DE}^{0}$ represents the energy density of dark energy at the present time. We note the Starobinsky f(R) model clearly has a larger dark energy density than ΛCDM does as $\rho_{DE}>\rho_{DE}^{0}$ in z≲4. In addition, the f(R) model in the closed universe (i.e., $\Omega_{K}<0$, K>0) contributes to a larger dark energy density in z≲4, which covers the dark energy dominant epoch. This result of enlarged dark energy can be seen from Equation (8) as 0<F<1 and K>0. Furthermore, we show in Figure 2 that $w_{DE}$ runs from the phantom phase ($w_{DE}<-1$) to the non-phantom phase ($w_{DE}>-1$) as *z* decreases, while it evolves faster in the closed universe. We note that $w_{DE}$ starts to oscillate in the region 10≳z≳4. The oscillation properties in the Starobinsky f(R) model are discussed in Reference [39].

Using

(19) $$\begin{array}{c} t_{age}=\frac{1}{H_{0}}\int_{0}^{1}\frac{da}{a\sqrt{\Omega_{m}a^{-3}+\Omega_{r}a^{-4}+\Omega_{K}a^{-2}+\Omega_{de}\left(a\right)}}, \end{array}$$

we have

(20) $$\begin{array}{cc} t_{age}^{open} & =13.946,\;14.005\;Gyr\;(\Omega_{K}=0.01) \end{array}$$

(21) $$\begin{array}{cc} t_{age}^{flat} & =13.984,\;14.049\;Gyr\;(\Omega_{K}=0) \end{array}$$

(22) $$\begin{array}{cc} t_{age}^{closed} & =14.021,\;14.094\;Gyr\;(\Omega_{K}=-0.01), \end{array}$$

for the Starobinsky f(R) and ΛCDM model, respectively. Note that the negative value of $\Omega_{K}$ in the closed universe will result in a larger $t_{age}$. However, the enlarged $\Omega_{DE}$ in f(R) gravity will compensate for its effect. Moreover, the bigger value of $t_{age}$ is related to the longer growth time of the large scale structure (LSS) as well as the larger matter density fluctuations.

### 3.2. Global Fitting Results

In this subsection, we constrain the cosmological parameters for the Starobinsky f(R) model with $\Omega_{K}$ set as a free parameter. We use the combination of datasets to break the geometrical degeneracy [40,41]. Explicitly, these datasets include CMB temperature and polarization angular power spectra from *Planck* 2018 with TT, TE, EE, low-*l* polarization, CMB lensing from SMICA [3,4,5,6], BAO observations form 6-degree Field Galaxy Survey (6dF) [7], SDSS DR7 Main Galaxy Sample (MGS) [42] and BOSS Data Release 12 (DR12) [43], and supernova (SN) data from the Pantheon compilation [44]. There are nine free parameters in our fitting of the Starobinsky f(R) model as we set the density parameter of curvature and the neutrino mass sum to be free, where the priors are listed in Table 1.

**Figure 3:**
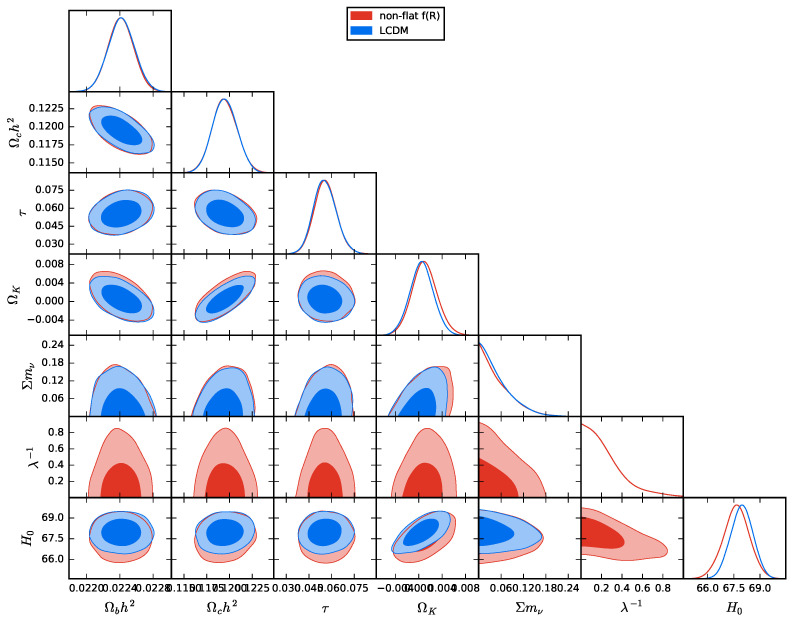
One and two-dimensional distributions of $\Omega_{b}^{0}h^{2}$, $\Omega_{c}^{0}h^{2}$, τ, $\Omega_{K}$, $\sum m_{\nu }$, $\lambda^{-1}$ and $H_{0}$ for the Starobinsky f(R) and ΛCDM models without the flatness assumption with the combined data of CMB, BAO and Pantheon data sets, where the contour lines represent 68% and 95% C.L., respectively.

The constraints on the cosmological parameters of the Starobinsky f(R) model without the flatness assumption with CMB+BAO+SN datasets are plotted in Figure 3 and listed in Table 2. We note that these constraints are barely distinguishable from those in ΛCDM. However, the model parameter $\lambda^{-1}$ in the Starobinsky f(R) model is relaxed as indicated in Figure 3. In particular, we find that $\lambda^{-1}<0.283$ at 68% C.L., which matches the previous study in Reference [38]. We also obtain the density parameter of curvature $\Omega_{K}=-0.00099_{-0.0042}^{+0.0044}$ at 95% C.L. for the Starobinsky f(R) model. Note that the flat ΛCDM model is recovered when $\lambda^{-1}=0$ and $\Omega_{K}=0$.

Our results also show that the neutrino mass sum is constrained to be $\Sigma m_{\nu }<0.137\;\left(0.132\right)$ for f(R) (ΛCDM), in which the value in f(R) is relaxed about 3.8% compared with that in ΛCDM. This phenomenon is caused by the shortened age of the universe in the Starobinsky f(R) model, which suppresses the matter density fluctuation as discussed in Reference [38]. We note that our fitting results give that $\chi^{2}=3821.72\;\left(3821.84\right)$ for f(R) (ΛCDM) with $\chi_{f(R)}^{2}≲\chi_{\Lambda CDM}^{2}$, indicating that the Starobinsky f(R) model can be a good candidate to describe the cosmological evolutions with $\Omega_{K}$ being a free parameter.

To compare Starobinsky f(R) gravity with ΛCDM for the best fitting results, we introduce the Akaike Information Criterion (AIC) [45], Bayesian Information Criterion (BIC) [46], and Deviance Information Criterion (DIC) [47]. The AIC, defined through the maximum likelihood $L_{max}$ under the Gaussian likelihood assumption and the number of model parameters, *d*, is given by

(23) $$\begin{array}{c} AIC=-2\;\ln\;L_{max}+2d=\chi_{min}^{2}+2d. \end{array}$$

The BIC is defined as

(24) $$\begin{array}{c} BIC=-2\;\ln\;L_{max}+d\ln N=\chi_{min}^{2}+d\ln N, \end{array}$$

where *N* is the number of data points. The DIC is determined by the quantities obtained from posterior distributions, written as

(25) $$\begin{array}{c} DIC=D\left(\bar{\theta }\right)+2\;p_{D}, \end{array}$$

where D(θ)=−2lnL(θ)+C with *C* as a constant, and $p_{D}$ is the effective number of parameters in the model.

Our results of the AIC, BIC and DIC from CMB+BAO+SN samples for the Starobinsky f(R) and ΛCDM models are summarized in Table 3, in which the differences between the criterions are found to be $\Delta AIC=AIC_{f(R)}-AIC_{\Lambda CDM}=1.88$, $\Delta BIC=BIC_{f(R)}-BIC_{\Lambda CDM}=8.07$, and $\Delta DIC=DIC_{f(R)}-DIC_{\Lambda CDM}=2.03$, respectively. It is clear that there is no preference between the two models [48] as (ΔAIC,ΔDIC)≲2. However, it would be evidence against the Starobinsky f(R) model as 6<ΔBIC<10 [49].

## 4. Conclusions

We have investigated the evolutions of the normalized effective dark energy density $\rho_{DE}/\rho_{DE}^{0}$ and equation of state $w_{DE}$ for the Starobinsky f(R) model in a non-flat universe. We have shown that the Starobinsky f(R) model in the closed universe contributes to a larger dark energy density and faster evolved dark energy equation of state. We have also given the constraints on the cosmological parameters in the Starobinsky f(R) model by modifying the CAMB and CosmoMC packages at the background level. Explicitly, we have obtained the parameters of the Starobinsky f(R) model and curvature density to be $\lambda^{-1}<0.283$ at 68% C.L. and $\Omega_{K}=-0.00099_{-0.0042}^{+0.0044}$ at 95% C.L., respectively. We have also found that the neutrino mass sum in f(R) is relaxed about 3.8% comparing with that in ΛCDM, which is caused by the shortened age of the universe that suppresses the matter density fluctuation in the Starobinsky f(R) model. Furthermore, the best-fitted $\chi^{2}$ values for the Starobinsky f(R) model are slightly less than that for the ΛCDM model, indicating that f(R) gravity is consistent with ΛCDM without the flatness assumption. We have also compared the AIC, BIC and DIC results of the two models. We have found that ΛCDM is slightly more preferable in terms of BIC, but such a conclusion cannot be made based on AIC and DIC. 

## Figures and Tables

**Table 1:** Priors of cosmological parameters for Starobinsky f(R) and ΛCDM models in the non-flat universe.

Parameter	Prior
f(R) model parameter $\lambda^{-1}$	$10^{-4}\leq \lambda^{-1}\leq 1$
Curvature parameter $\Omega_{K}$	$-0.1\leq \Omega_{K}\leq 0.1$
Baryon density	$0.5\leq 100\Omega_{b}h^{2}\leq 10$
CDM density	$0.1\leq 100\Omega_{c}h^{2}\leq 99$
Optical depth	0.01≤τ≤0.8
Neutrino mass sum	$0\leq \Sigma m_{\nu }\leq 2$ eV
$\frac{\mathrm{Sound}\;\mathrm{horizon}}{\mathrm{Angular}\;\mathrm{diameter}\;\mathrm{distance}}$	$0.5\leq 100\theta_{MC}\leq 10$
Scalar power spectrum amplitude	$1.61\leq \ln\left(10^{10}A_{s}\right)\leq 3.91$
Spectral index	$0.8\leq n_{s}\leq 1.2$

**Table 2:** Fitting results in Starobinsky f(R) and ΛCDM models without the flatness assumption with the CMB, BAO and Pantheon data sets, where the cosmological parameters and the model parameter $\lambda^{-1}$ are constrained at 95% C.L. and 68% C.L., respectively.

Parameter	Starobinsky f(R)	ΛCDM
$\Omega_{b}h^{2}$	$0.02241_{-0.00030}^{+0.00030}$	$0.02242_{-0.00031}^{+0.00031}$
$\Omega_{c}h^{2}$	$0.1195_{-0.0027}^{+0.0028}$	$0.1195_{-0.0026}^{+0.0027}$
$100\theta_{\mathrm{MC}}$	$1.04097_{-0.00063}^{+0.00062}$	$1.04100_{-0.00062}^{+0.00059}$
τ	$0.056_{-0.014}^{+0.016}$	$0.056_{-0.014}^{+0.016}$
$\Omega_{K}$	$0.00099_{-0.0042}^{+0.0044}$	$0.0005_{-0.0040}^{+0.0040}$
$\Sigma m_{\nu }$	<0.137 eV	<0.132 eV
$\ln(10^{10}A_{s})$	$3.047_{-0.028}^{+0.030}$	$3.046_{-0.027}^{+0.031}$
$n_{s}$	$0.9664_{-0.0089}^{+0.0087}$	$0.9666_{-0.0084}^{+0.0082}$
$\lambda^{-1}$	<0.283	−
$H_{0}$	$67.6_{-1.5}^{+1.5}$	$68.0_{-1.2}^{+1.2}$
$\sigma_{8}$	$0.811_{-0.021}^{+0.020}$	$0.814_{-0.019}^{+0.018}$
Age/Gyr	$13.74_{-0.17}^{+0.16}$	$13.76_{-0.15}^{+0.15}$
$\chi_{\mathrm{best}-\mathrm{fit}}^{2}$	3821.72	3821.84

**Table 3:** The results of AIC, BIC and DIC computed from the sample we used for both ΛCDM and exponential f(R) models, where $\Delta AIC=AIC_{f(R)}-AIC_{\Lambda CDM}$, $\Delta BIC=BIC_{f(R)}-BIC_{\Lambda CDM}$, and $\Delta DIC=DIC_{f(R)}-DIC_{\Lambda CDM}$.

Model	$\chi_{\min}^{2}$	AIC	ΔAIC	BIC	ΔBIC	DIC	ΔDIC
ΛCDM	3821.84	3837.84	0	3887.35	0	3850.38	0
Starobinsky f(R)	3821.72	3839.72	1.88	3895.42	8.07	3852.41	2.03

## Data Availability

The data for BAO and Pantheon presented in this study are available in [7,42,43,44]. Publicly available datasets were analyzed in this study. The data for CMB can be found here: http://pla.esac.esa.int/pla/#cosmology, accessed on 28 September 2021.
